# A one-step low-cost molecular test for SARS-CoV-2 detection suitable for community testing using minimally processed saliva

**DOI:** 10.1093/biomethods/bpae035

**Published:** 2024-05-22

**Authors:** Sofia M da Silva, Catarina Amaral, Cláudia Malta-Luís, Diana Grilo, Américo G Duarte, Inês Morais, Gonçalo Afonso, Nuno Faria, Wilson Antunes, Inês Gomes, Raquel Sá-Leão, Maria Miragaia, Mónica Serrano, Catarina Pimentel

**Affiliations:** Instituto de Tecnologia Química e Biológica António Xavier, Universidade Nova de Lisboa, Oeiras, 2780-157, Portugal; Instituto de Tecnologia Química e Biológica António Xavier, Universidade Nova de Lisboa, Oeiras, 2780-157, Portugal; Instituto de Tecnologia Química e Biológica António Xavier, Universidade Nova de Lisboa, Oeiras, 2780-157, Portugal; Instituto de Tecnologia Química e Biológica António Xavier, Universidade Nova de Lisboa, Oeiras, 2780-157, Portugal; Instituto de Tecnologia Química e Biológica António Xavier, Universidade Nova de Lisboa, Oeiras, 2780-157, Portugal; Instituto de Tecnologia Química e Biológica António Xavier, Universidade Nova de Lisboa, Oeiras, 2780-157, Portugal; Instituto de Tecnologia Química e Biológica António Xavier, Universidade Nova de Lisboa, Oeiras, 2780-157, Portugal; Instituto de Tecnologia Química e Biológica António Xavier, Universidade Nova de Lisboa, Oeiras, 2780-157, Portugal; Centro de Investigação da Academia Militar (CINAMIL), Unidade Militar Laboratorial de Defesa Biológica e Química (UMLDBQ), Av. Dr Alfredo Bensaúde, Lisboa, 1849-012, Portugal; Centro de Investigação da Academia Militar (CINAMIL), Unidade Militar Laboratorial de Defesa Biológica e Química (UMLDBQ), Av. Dr Alfredo Bensaúde, Lisboa, 1849-012, Portugal; Instituto de Tecnologia Química e Biológica António Xavier, Universidade Nova de Lisboa, Oeiras, 2780-157, Portugal; Instituto de Tecnologia Química e Biológica António Xavier, Universidade Nova de Lisboa, Oeiras, 2780-157, Portugal; Instituto de Tecnologia Química e Biológica António Xavier, Universidade Nova de Lisboa, Oeiras, 2780-157, Portugal; Instituto de Tecnologia Química e Biológica António Xavier, Universidade Nova de Lisboa, Oeiras, 2780-157, Portugal

**Keywords:** COVID-19, diagnostics, RT-PCR SYBR Green, saliva test

## Abstract

The gold standard for coronavirus disease 2019 diagnostic testing relies on RNA extraction from naso/oropharyngeal swab followed by amplification through reverse transcription-polymerase chain reaction (RT-PCR) with fluorogenic probes. While the test is extremely sensitive and specific, its high cost and the potential discomfort associated with specimen collection made it suboptimal for public health screening purposes. In this study, we developed an equally reliable, but cheaper and less invasive alternative test based on a one-step RT-PCR with the DNA-intercalating dye SYBR Green, which enables the detection of severe acute respiratory syndrome coronavirus 2 (SARS-CoV-2) directly from saliva samples or RNA isolated from nasopharyngeal (NP) swabs. Importantly, we found that this type of testing can be fine-tuned to discriminate SARS-CoV-2 variants of concern. The saliva RT-PCR SYBR Green test was successfully used in a mass-screening initiative targeting nearly 4500 asymptomatic children under the age of 12. Testing was performed at a reasonable cost, and in some cases, the saliva test outperformed NP rapid antigen tests in identifying infected children. Whole genome sequencing revealed that the antigen testing failure could not be attributed to a specific lineage of SARS-CoV-2. Overall, this work strongly supports the view that RT-PCR saliva tests based on DNA-intercalating dyes represent a powerful strategy for community screening of SARS-CoV-2. The tests can be easily applied to other infectious agents and, therefore, constitute a powerful resource for an effective response to future pandemics.

## Introduction

One lesson from coronavirus disease 2019 (COVID-19) is that timely and accurate diagnosis, along with a robust testing capacity and effective contact tracing, is crucial in controlling the early stages of a pandemic. In fact, COVID-19 testing played a pivotal role in providing a clear, real-time understanding of the global infection rate. This valuable information has been instrumental in guiding the implementation of measures by public health authorities [1, 2]. As a result, many countries recognized the importance of testing and made significant investments to enhance their testing capacity during that period [3, 4]. Testing primarily relied on the gold standard test for COVID-19 diagnosis, which involves real-time reverse transcription-polymerase chain reaction (RT-PCR) with fluorogenic probes. This test amplifies and detects the genetic material of severe acute respiratory syndrome coronavirus 2 (SARS-CoV-2), obtained from the nasopharyngeal (NP) or oropharyngeal fluid through swab collection performed by a healthcare professional.

Although the gold standard test for COVID-19 is highly sensitive and specific, it presents several challenges. It is expensive, has long turnaround times, requires skilled personnel throughout the process, from sample collection to result delivery, and the NP or oropharyngeal swab can be uncomfortable. These factors limited its suitability for widespread public health testing. As a result, there was a push to develop cheaper and faster alternative nucleic acid amplification tests that could reliably detect SARS-CoV-2 in clinical specimens other than NP or oropharyngeal swabs, such as saliva [5–7]. Saliva offers the advantage of easy self-collection, eliminating the requirement for healthcare professionals at collection points, which not only reduces testing costs but also lowers the risk of transmission. Despite some controversy [8], numerous studies have demonstrated that saliva provides a very good diagnostic performance and is a clinically acceptable alternative to NP or oropharyngeal samples in detecting SARS-CoV-2 [9–14]. Moreover, saliva can serve as a route for infection [13] and saliva from asymptomatic individuals with COVID-19 has demonstrated potential for viral transmission [13].

Several strategies have been attempted to reduce the costs associated with COVID-19 diagnosis. One promising and cost-effective approach, involved the development and testing of RT-PCR protocols that utilize saliva, pooled samples, or DNA-intercalating dyes instead of fluorogenic probes [5, 15–17]. DNA-intercalating dyes, such as SYBR Green, are particularly appealing due to their lower cost compared to fluorogenic probes. In addition, assays using DNA-intercalating dyes are easier and faster to set up, and they are commonly employed in non-clinical laboratories. These features make diagnostic tests based on real-time PCR with DNA-intercalating dyes a promising strategy for decentralizing diagnosis and increasing the testing capacity and autonomy of countries during a pandemic.

Previous studies have demonstrated that singleplex or multiplex SYBR Green-based one-step or two-step RT-PCR tests designed for COVID-19 exhibit excellent sensitivity [16, 18–27]. One advantage of these tests, as opposed to RT-PCR with fluorogenic probes, is that they allow for assessment of the amplification specificity by simply examining the amplicon melting curves at the end of the PCR [28]. While most SYBR Green-based RT-PCR assays have been established for naso or oropharyngeal specimens [16, 18–27], a handful of studies showed promising evidence for using saliva samples in these tests [17, 29–31]. However, all the protocols described thus far require prior viral RNA isolation, which increases the costs and time needed for testing.

In this study, we have successfully developed a one-step singleplex SYBR Green-based RT-PCR test for the accurate detection of SARS-CoV-2 directly from saliva clinical samples that eliminates the need for RNA extraction. The test can also be applied to RNA samples isolated from naso or oropharyngeal swabs, offering great flexibility in testing options. Our study also highlights the effectiveness and practicality of using the direct saliva test in a community setting. The results suggest that implementing this type of testing has the potential to significantly increase our testing capacity and improve our ability to manage and control infectious outbreaks more effectively in the future.

## Materials and methods

### Sample collection, processing, and storage

NP specimens were collected at Hospital das Forças Armadas or at the testing centre of Fundição de Oeiras and processed at Laboratório de Bromatologia e Defesa Biológica (Unidade Militar Laboratorial de Defesa Biológica e Química), or at ITQB NOVA, respectively. NP samples were inactivated by incubation at 95°C for 5 min.

Saliva specimens were self-collected at the Hospital and at the testing centre of Fundição de Oeiras (testing optimization), or at home (community testing) and processed at ITQB NOVA. Saliva samples (approximately 1 ml) were self-collected by spitting into sterile tubes (50 ml or 1.5 ml). In order to optimize saliva testing, NP swab-matched samples were collected simultaneously. Participants were instructed not to eat or drink prior to testing. After collection, saliva samples were either stored at 4°C for 2–4 days or processed immediately. The saliva specimens were inactivated at 95°C for 30 min, centrifuged at 5000 g for 5 min and 200 µl of the supernatant were diluted in 10x TE (final concentration, 1×) and frozen at −80°C until analysis [32]. The remaining non-diluted supernatant was also frozen for storage.

### RNA extraction from clinical samples

Total viral RNA was extracted from 140 µl of deactivated samples (NP or non-diluted saliva supernatants) using Viral RNA Mini Kit (QIAGEN) and eluted in 60 µl of RNAse free water.

### SARS-CoV-2 RNA standard

SARS-CoV-2 RNA standard was prepared by amplifying the N gene from the plasmid 2019-nCoV_N_Positive Control (Integrated DNA Technologies) with a T7-promoter-containing primer (5′-TAATACGACTCACTATAGGatgtctgataatggaccccaaaa-3) and the reverse primer (5′-ttaggcctgagttgagtcagc-3′). The product was transcribed *in vitro* using HiScribe T7 High Yield RNA Synthesis Kit (NEB) following the manufacturer’s instructions. Template DNA was removed using Turbo DNase (Invitrogen) and RNA was then purified using RNeasy Mini Kit (Qiagen). Standard RNA copy numbers were calculated based on the RNA concentration measured using Epoch Take3 spectrophotometer (Biotek).

### RT-PCR with SYBR Green

One-step qRT-PCR was performed with SYBR FAST One-Step qRT-PCR Master Mix Kit (KAPA Biosystems) according to the manufacturer’s instructions. An aliquot of 4 µl of sample (saliva or RNA from NP swabs) was added to the master mix to a final volume of 10 µl in a 96-well PCR plate. RT-PCR was performed in the LightCycler 480 (Roche) or the LightCycler 96 SW 1.1, using the following settings: reverse transcription step (50°C for 10 min and 95°C for 3 min), amplification step (40 or 33 cycles of 94°C for 30 s; 58°C for 30 s and 72°C for 5 s), melting curve step (95°C for 5 s; 65°C for 1 min and 97°C in continuous mode) and final cooling step (40°C, 30 s). The N gene was amplified with the primers 2019-nCoV N1-F (5′-GACCCCAAAATCAGCGAAAT-3′) and 2019-nCoV_N1-R (5′-TCTGGTTACTGCCAGTTGAATCTG-3′) [33]. The LC480 Instrument Software version 1.5.0 was used to calculate the cycle threshold (CT) using the *2nd derivative method* for amplification reactions with 40 cycles and the *Fit points method* for amplification reactions with 33 cycles. T_m_ calling was performed on all reactions to confirm the presence of the specific amplification product and to dismiss any false positives. Samples exhibiting a clear amplification and melting curves, with Tm matching +/− 1°C that of the positive control (25 copies/rnx) were considered positive.

The analytical limit of detection (LoD) was defined as the lowest concentration of IVT N-gene RNA at which at least 19 out of 20 (95%) replicates tested positive [34]. For this, a known number of copies of the IVT N-gene RNA either diluted in water or spiked into saliva, was used.

### RT-PCR with fluorogenic probes (gold standard test)

For samples collected at Hospital das Forças Armadas, SARS-CoV-2 N gene and an internal control (RNase P) were amplified by RT-PCR using the TaqMan 2019-nCoV Assay Kit v1 (Thermofisher) with TaqMan Fast Virus 1-step Master Mix (Thermofisher) and the CFX96 thermocycler (BioRad), according to the manufacturer’s instructions For samples collected at the testing centre of Fundição de Oeiras, SARS-CoV-2 ORF1ab, N gene and E gene were amplified by RT-PCR using the 2019-nCoV Real-time fluorescent RT-PCR kit (Fosun) and the LM2912 thermocycler (Fosun Diagnostics), according to the manufacturer’s instructions.

### Expression and purification of Taq DNA polymerase and MashUP reverse transcriptase (RT)

The *Thermus aquaticus* (Taq) DNA polymerase gene cloned into the pUC18 DNA vector was used to transform *Escherichia coli* DH5α competent cells, which were then induced with 0.5 mM IPTG for 18 h at 37°C, 180 rpm. Cells were harvested by centrifugation at 4°C and resuspended in a buffer solution containing 50 mM Tris–HCl pH 7.9, 50 mM glucose, and 10 mM EDTA. After the addition of lysozyme (4 mg/ml), the cell suspension was incubated at room temperature for 15 min, diluted twice with a solution containing 10 mM Tris–HCl pH 7.9, 50 mM KCl, 1 mM EDTA, 0.5% (v/v) Tween 20, 0.5% (v/v) Nonidet P40 and further incubated at 75°C with agitation for 1 h, to precipitate *E. coli* proteins. Precipitated proteins were separated by centrifugation at 12 000 g for 20 minutes at 4°C and the supernatant was subjected to buffer exchange (10 mM Tris–HCl pH 8, 100 mM NaCl, 0.1 mM EDTA, 0.5 mM DTT, 1% (v/v) Triton X-100, 50% (v/v) glycerol). Protein aliquots were flash frozen in liquid nitrogen and stored at −80°C until ready for use. The MashUp RT was obtained from a plasmid kindly provided by https://pipettejockey.com, according to the protocol detailed in [32].

### In-house one-step EvaGreen-based RT-PCR assay

One step RT-PCR was performed with 1x EvaGreen dye (Biotium), the in-house made enzymes Taq DNA polymerase and MashUp RT (0.5 µl each), 1x reaction buffer (NZYTech), 2.5 mM MgCl_2_ (NZYTech), 12.5 mM KCl and 0.5 mM dNTPs (NZYTech). The N gene was amplified using 200 nM of the primer pair 2019-nCoV_N1-F and 2019-nCoV_N1-R [5]. An aliquot of 4 µl of RNA were added to the reaction mix to a final volume of 20 µl in a 96-well PCR plate. The analysis was performed in the LightCycler 480 (Roche) using the following settings: reverse transcription step (50°C for 10 min and 95°C for 3 min), amplification step (33 cycles of 94°C for 30 s, 60°C for 30 s and 72°C for 5 s), melting curve (95°C for 5 s; 65°C for 1 min and 97°C in continuous mode) and final cooling step (40°C, 30 s). CT determination and Tm calling was performed following the methods described above.

### Vectors and primers for VOC detection

Two plasmids were synthesized by GenScript, one containing the sequence of the original SARS-CoV-2 strain (pWT), and other carrying the specific mutation Δ3675-3677 in ORF1a (pVOCα) that mimics variant B1.1.7. One-step RT-PCR was performed with SYBR FAST One-Step qRT-PCR Master Mix Kit (KAPA Biosystems) according to the manufacturer’s instructions. An aliquot of 4 µl of each DNA plasmid (pWT or pVOCα) was added to the master mix to a final volume of 10 µl in a 96-well PCR plate. The forward (Fw_Orf_mis 5′-GGTTGGATATGGTTGATACTAGTTTGCA-3′) and reverse (Rv_Orf 5′-GTTCTTGCTGTCATAAGGATTAGTAAC-3′) primers were used at a final concentration of 100 nM. The analysis was performed in the LightCycler 480 (Roche) using the following settings: reverse transcription step (50°C, 10 min and 95°C, 3 min), amplification step (35 cycles of 94°C, 30 s; 62°C, 30 s and 72°C, 5 s), melting curve step (95°C for 5 s; 65°C for 1 min and 97°C in continuous mode) and a final cooling step (40°C, 30 s). CT determination and Tm calling was performed following the methods described above. The two RNA samples analysed were obtained from patients who had confirmed infections with the SARS-CoV-2 variants Alpha and Omicron BA.1. Variant identification was previously confirmed through whole genome sequencing, using long reads from Oxford Nanopore Technologies (see below).

### Community screening

From January 2022 to March 2022, in partnership with the Municipality of Oeiras, Portugal, the ITQB NOVA provided free saliva testing using the SYBR Green-based one-step RT-PCR to volunteer students (aged 3–11 years) from all public schools in Oeiras. Circa 9500 saliva collection kits were provided and 4445 tests were completed. Students were instructed to collect the saliva sample in the morning on a pre-defined day before eating, drinking, and brushing teeth. The samples were kept at 4°C, promptly transported to ITQB NOVA, and analysed on the same day of collection. Confirmatory standard RT-PCR tests with NP sampling were performed on participants with positive saliva tests, typically 24–48 h after the notification of the result. Students who had tested positive for COVID-19 in the 3 months prior to the screening initiative were excluded from the study.

### Genome sequencing and bioinformatics analysis

RNA samples were directly used for genome sequencing following the classic PCR tilling of SARS-CoV-2 virus protocol, from Oxford Nanopore Technologies, and further described in the ARTIC network protocol (https://artic.network/ncov-2019; https://www.protocols.io/view/ncov-2019-sequencing-protocol-bbmuik6w). This constitutes an amplicon-based whole-genome amplification strategy using tiled, multiplexed primers [35]. In brief, SARS-CoV-2 gRNA was reverse transcribed and the obtained cDNA was amplified (400 bp amplicons), recurring to two separate pools of tiling primers (ARTIC V3), using the NEBNext ARTIC SARS-CoV-2 Companion Kit (Oxford Nanopore Technologies, Oxford, UK). The generated pools of multiplexed amplicons were combined, for each sample, purified and prepared for barcoding (Native Barcoding Expansion, EXP-NBD104), to allow multiplex sequencing. Additionally, sequencing adapters were ligated to the pooled barcoded samples, according to manufacturers’ instructions. Sequencing libraries were sequenced in multiplex on one flow cell (R9.4.1) using a MinION Mk1C device, according to the settings described in the classic PCR tilling of SARS-CoV-2 virus protocol. MinKNOW (version 21.11.) software was used for basecalling (min Qscore 8) and reads demultiplexing, so downstream bioinformatics analysis could be performed. All bioinformatics analyses were performed using EPI2ME (version 3.4.2) and the Fastq QC + ARTIC + NextClade workflow. A consensus sequence was obtained using the genome sequence of SARS-CoV-2 Wuhan-Hu-1/2019 virus (GenBank accession number MN908947) as reference [36]. All the SARS-CoV-2 genome sequences included in the analysis had >70% of the genome covered by at least 20-fold, presenting an overall quality score of 11.5; regions with a depth of coverage below this threshold were automatically marked as ambiguous and undefined bases ‘N’ were introduced in the consensus sequence. Clade and lineage assignments were performed using Nextclade (version 2.4.1) (https://clades.nextstrain.org/) (accessed on 04 August 2022).

## Results

### Sensitivity and specificity of an SYBR Green-based one-step RT-PCR assay in detecting SARS-CoV-2 in NP samples

**Figure 1.**
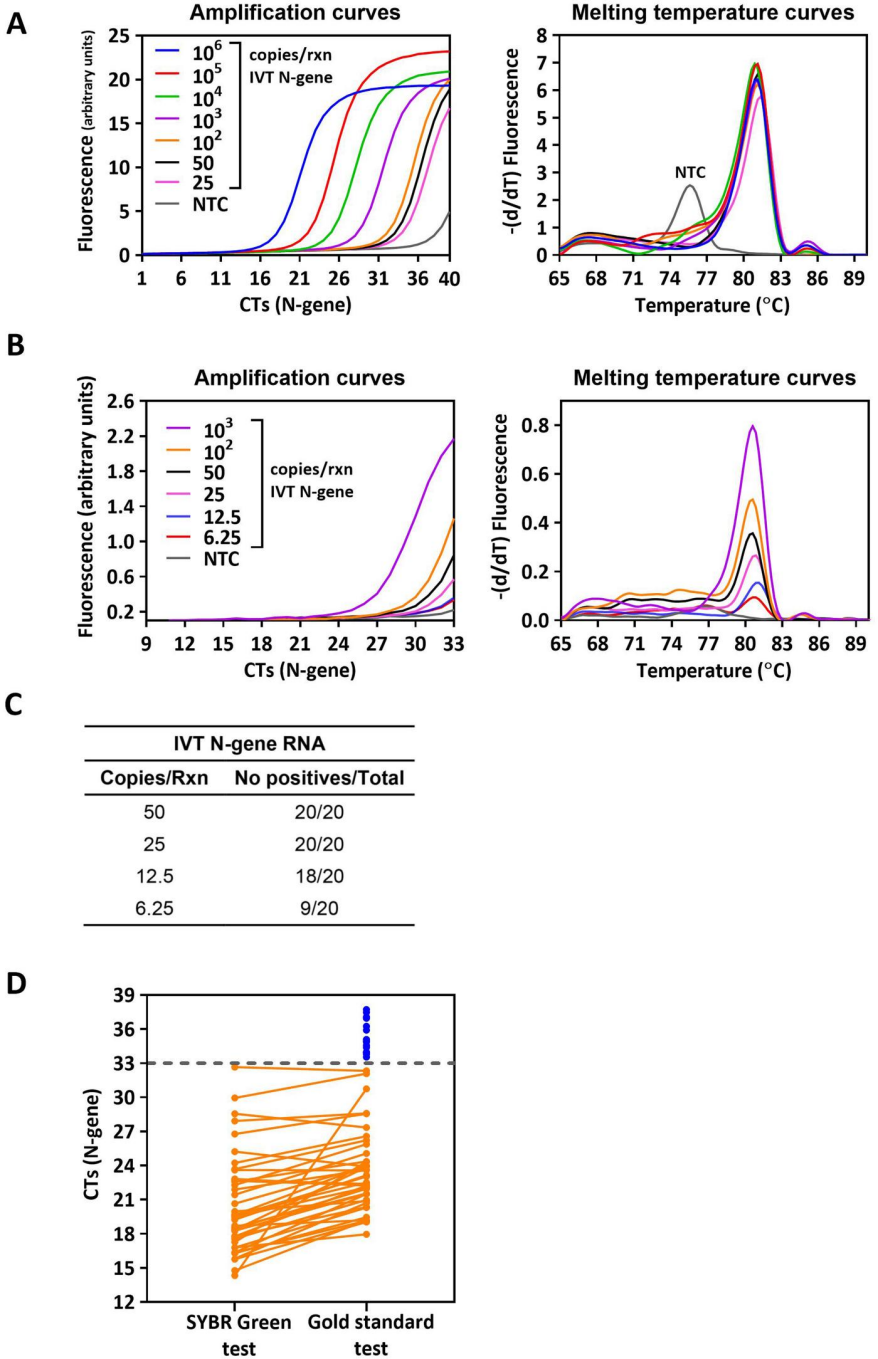
Sensitivity of the SYBR Green RT-PCR assay for SARS CoV-2 detection. Amplification and melting temperature profiles obtained with the SYBR Green RT-PCR assay after (A) 40 or (B) 33 PCR cycles. (C) LoD of the assay after 33 PCR cycles. (D) Comparison of the CT values obtained with the SYBR Green test and the gold standard test with fluorogenic probes; 56 RNA samples were extracted from NP swab samples of SARS-CoV-2 infected patients and amplified using both strategies. The blue dots indicate RNA samples from infected individuals that did not amplify with the SYBR Green test. NTC, no template control.

The costs associated with the reference diagnostic test for COVID-19 have made it unaffordable for countries with limited resources and inadequate for periodic mass screening purposes. The interest in overcoming such limitation prompted us to test an alternative RT-PCR protocol that uses inexpensive DNA-intercalating dyes instead of costly fluorogenic probes. For the PCR reaction, we chose a singleplex strategy to amplify a fragment of the viral RNA (N gene) using the N1 primer set designed and proved to be specific for SARS-CoV-2 by other authors [5]. We first amplified serial dilutions of the *in vitro* transcribed SARS-CoV-2 N gene (IVT N-gene) in order to estimate the number of genomic copies of the virus that could be detected. However, analysis of the amplification and amplicon melting temperature curves indicated the presence of unspecific amplifications in the no template control (NTC) after 40 PCR cycles (Fig. 1A). This was likely due to the formation of primer dimers, which could interfere with the accurate identification of positive samples. In order to mitigate this issue, we adjusted the number of PCR cycles to 33. We reasoned that while this adjustment may slightly decrease the sensitivity of the assay, it would greatly enhance the specificity. With this modified setup, we successfully eliminated non-specific amplifications (Fig. 1B and Supplementary Fig. S1), and we were able to reliably and consistently detect as few as 25 copies of the IVT N-gene per reaction (Fig. 1C).

We then used a set of 104 NP clinical samples to evaluate whether the SYBR Green-based RT-PCR could accurately detect SARS-CoV-2 in the NP fluid of infected individuals. The test was benchmarked against the gold standard for COVID‐19 diagnosis (RT-PCR with fluorogenic hybridization probes), using RNA isolated from those NP samples. For the majority of positive samples, we observed a close correlation between the CT values obtained from both the SYBR Green-based test and the gold standard test (Fig. 1D). For samples with CTs ≤ 33, the SYBR Green test showed excellent agreement (100%) with the gold standard (41 samples). As expected, for samples with CT values greater than or equal to 33 (15 samples), the SYBR Green assay failed to identify them as positive cases (Fig. 1D). Samples that were determined to be negative by the gold standard test (48 samples) also yielded negative results in the SYBR Green assay when subjected to 33 cycles of amplification. This indicates a high specificity of the SYBR Green assay under these conditions.

Overall, these results indicate that the SYBR Green-based one-step RT-PCR assay reliably detects medium to high viral loads in RNA isolated from NP swabs.

In a previous study, we presented a protocol for producing a reverse transcriptase, which we used in an in-house made RT-LAMP assay tailored for COVID-19 [32]. Here we have combined this enzyme with a newly produced Taq DNA polymerase and established a simple in-house made one-step RT-PCR test for detecting SARS-CoV-2. Following an optimization process involving fine-tuning of several reaction parameters (Supplementary Fig. S2), we decided to utilize the intercalating EvaGreen dye for the purpose of detection. With this assay, we were able to detect down to 50 copies of viral RNA per reaction (Supplementary Fig. S3A). Although the test exhibited lower sensitivity than the SYBR Green-based RT-PCR test, it reliably detected the genetic material of SARS-CoV-2 in RNA samples isolated from NP swabs (Supplementary Fig. S3B).

### The SYBR Green one-step RT-PCR assay can be used to detect variants of concern

**Figure 2.**
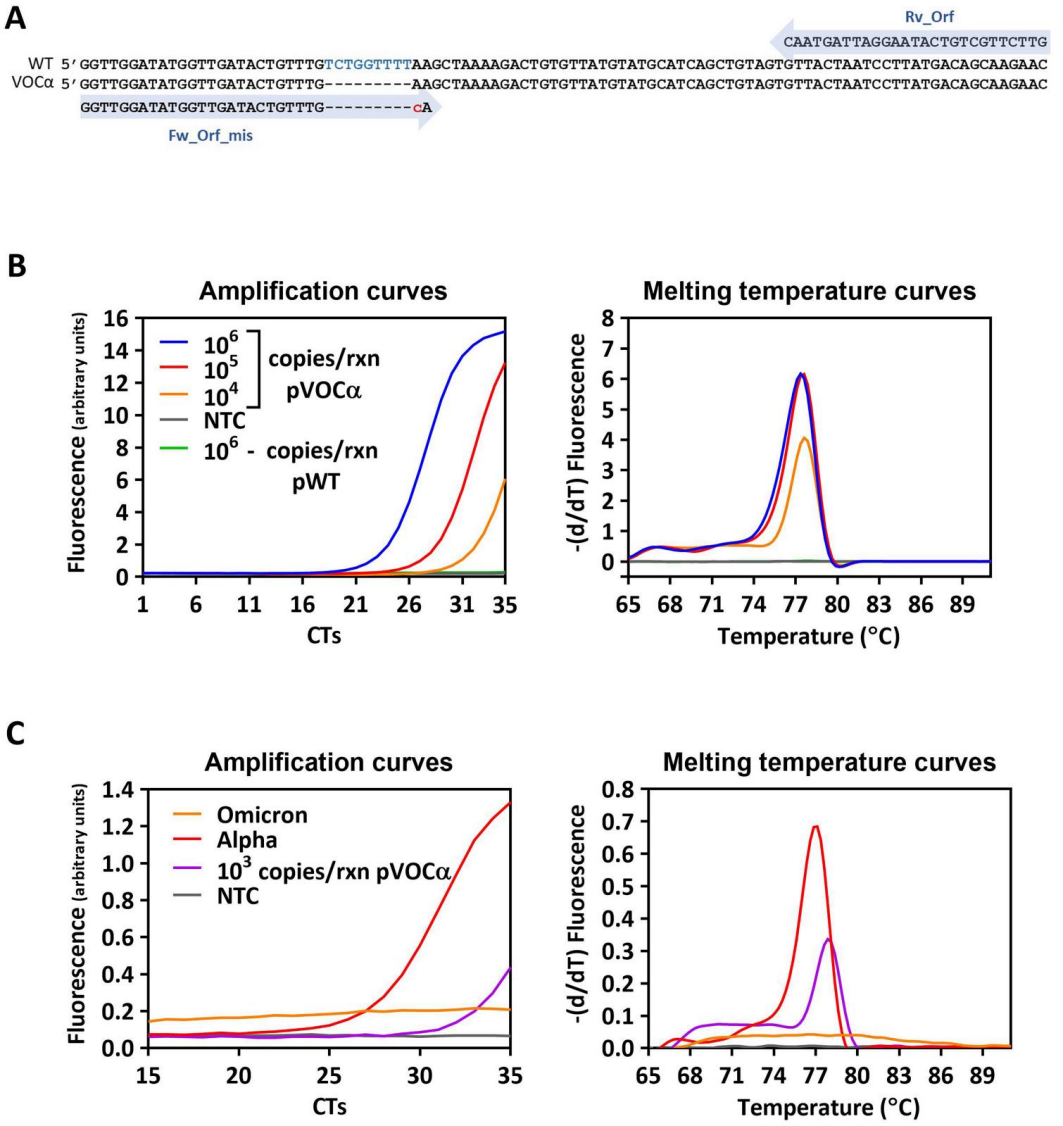
Performance of the SYBR Green RT-PCR assay in detecting SARS CoV-2 VOCα. (A) Specific primers designed to detect the ORF1a Δ3675-3677 mutation. Amplification plots for the SYBR Green RT-PCR assay using (B) known amounts of the vector carrying the mutation (pVOCα) or the original sequence (pWT) and (C) RNA extracted from NP swabs of SARS CoV-2 infected patients. NTC, no template control.

In late 2020, the emergence of SARS-CoV-2 variants that posed an increased threat to public health [37, 38] urged global monitoring of variants of concern (VOCs) in order to control their spread, provide information to decision-makers, and implement appropriate measures. Mass testing played a crucial role in that scenario. However, VOC identification primarily relied on expensive genome sequencing methods, which not only added to the economic burden imposed by the pandemic but also restricted VOC identification in low-resource settings. Here, we conducted experiments to determine whether our simple and cost-effective SYBR Green-based RT-PCR assay could be utilized for VOC detection. We employed a strategy that targets fingerprint mutations specific to those variants. To demonstrate the proof-of-concept, our focus was on a specific region of the ORF1a gene of SARS-CoV-2 that exhibited a deletion in the Alpha variant’s genome (Δ3675-3677) [39]. Inspired by the allele-specific PCR method [40], we designed an oligonucleotide primer (Fw_Orf_mis, Fig. 2A) whose 3′ end perfectly matched the sequence of the Alpha variant (VOCα), but not that of the original wild-type strain (Wuhan-Hu1 strain, WT) due to the Δ3675-3677 deletion in the former. To further strengthen variant-specific primer hybridization, an additional mismatch was introduced at the 3′ end of the primer (Fig. 2A).

We next constructed two plasmids, pVOCα and pWT, each carrying a fragment of the ORF1a region with or without the deletion Δ3675–3677, respectively. Each plasmid was used as a DNA template in the SYBR Green-based RT-PCR assay to evaluate its capacity to distinguish different SARS-CoV-2 variants. Amplification conditions were first optimized by testing several primer concentrations, different annealing temperatures, and varying the number of amplification cycles (data not shown). After determining the optimal conditions, we performed amplification on serial dilutions of pVOCα and pWT. Notably, robust detection was achieved for all dilutions of pVOCα, while pWT remained undetectable (Fig. 2B). Lastly, to eliminate the possibility of mis-hybridization within the genome of SARS-Cov-2, we assessed the performance of the assay using two real-life RNA samples isolated from patients infected with the SARS-CoV-2 variants Alpha and Omicron BA.1. Amplification was observed only in the sample containing the Alpha variant (Fig. 2C) since the specific deletion (Δ3675–3677) targeted by the assay is absent in Omicron BA.1 [41]. As expected, the N1 primer set (Fig. 1) amplifies both sample variants (Supplementary Fig. S4).

### The SYBR Green-based one-step RT-PCR test demonstrates great analytical and clinical sensitivity in directly detecting SARS-CoV-2 from saliva samples

The advantages of using saliva samples instead of NP swabs for diagnosing COVID-19 have prompted us to investigate whether the one-step SYBR Green-based assay could be employed for identifying SARS-CoV-2 in saliva without the need for prior RNA extraction. Apart from offering a simpler and less invasive sample collection procedure, such a test would expedite the time to results and further reduce diagnostic costs. Prior to amplification, the samples underwent a straightforward pre-treatment process, which involved viral inactivation by heating at 95°C for 30 min and addition of TE buffer. We have preciously demonstrated that this procedure enables successful amplification of SARS-CoV-2 genetic material from saliva specimens [32]. Importantly, the tubes containing the saliva samples remained sealed following self-collection and throughout the entire inactivation process, ensuring the safety of the operating personnel.

**Figure 3.**
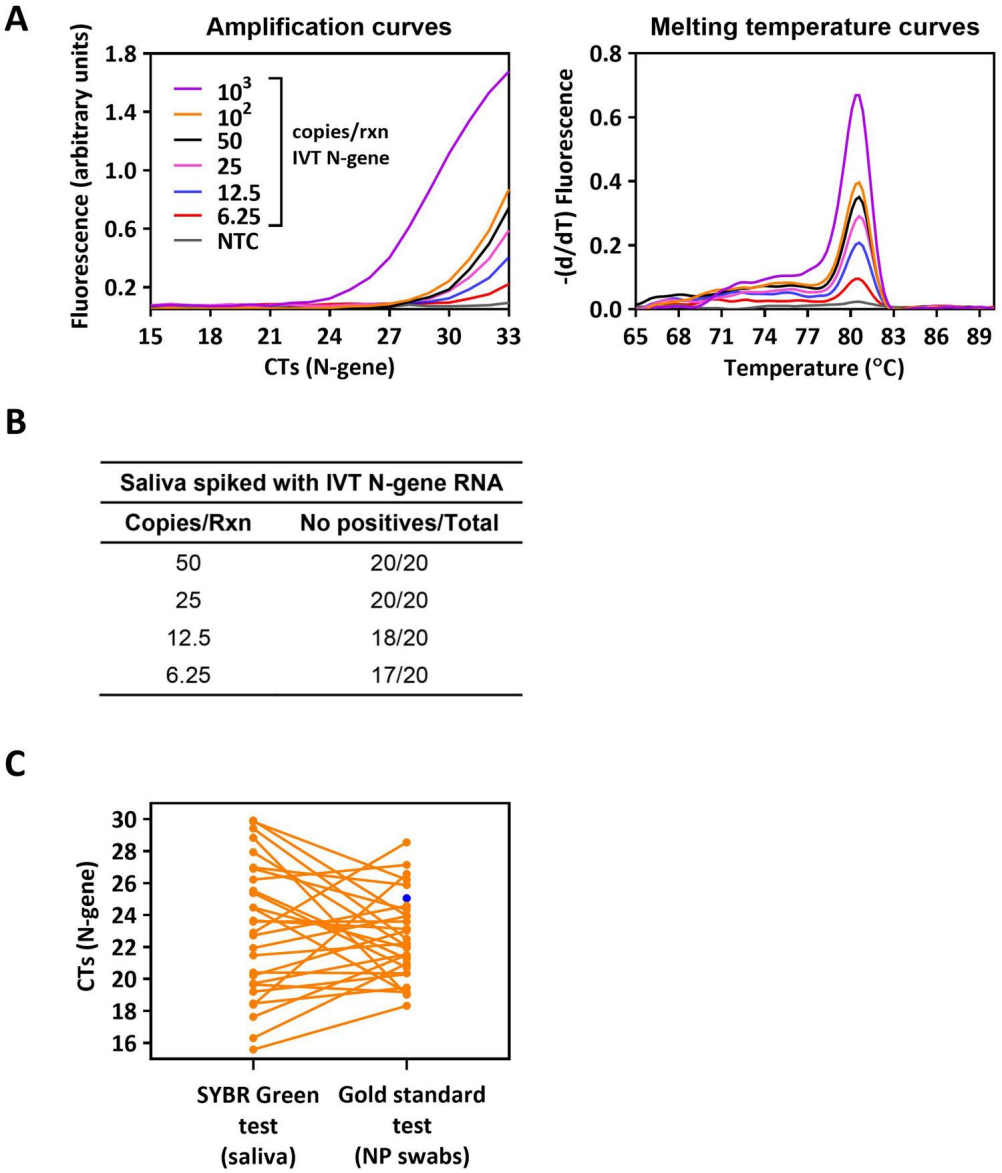
Sensitivity of the saliva SYBR Green RT-PCR test. (A) Amplification and melting curves obtained after the direct amplification of saliva samples spiked with a known copy number of the IVT N-gene. (B) Analytical LoD of the assay. (C) Scheme of the experimental layout used in (D) created in Biorender.com. (D) Comparison of the CT values obtained after amplification of paired samples (i.e. RNA extracted from NP swabs and saliva samples from the same individual) using the SYBR Green (saliva samples) and the RT-PCR gold standard (NP swabs) tests. The blue circle pinpoints the sample, for which the results of both tests disagree. NTC—no template control.

The analytical sensitivity of the SYBR Green-based RT-PCR saliva test was assessed by spiking saliva samples obtained from healthy donors (confirmed by the gold standard) with different amounts of the IVT N-gene. As described above, to decrease nonspecific amplification we defined a threshold of 33 cycles in the RT-PCR reaction. The analysis of the amplification and melting temperature curves (Fig. 3A) revealed clear profiles, and the test was able to detect as low as 25 copies/reaction of viral RNA (Fig. 3B).

We also evaluated the sensitivity and specificity of the saliva test in detecting the genetic material of SARS-CoV-2 from clinical samples. We conducted an analysis on paired NP swab and saliva samples from 129 individuals using both the gold standard RT-PCR (NP swab) and the SYBR Green-based RT-PCR (saliva) tests, with or without prior RNA extraction, respectively (Fig. 3C). According to the gold standard, 29 samples were positive and 100 were negative for SARS-CoV-2 infection, with the CTs of the former varying between 18.3 and 26.6. The SYBR Green-based RT-PCR successfully detected viral genetic material directly from the saliva samples, that is, without the need for RNA pre-extraction, in all but one positive sample (Fig. 3D). All 100 samples scored as negative by the gold standard test were also identified as negative by the SYBR Green-based RT-PCR, demonstrating the excellent specificity of the saliva test (Table 1). By eliminating the RNA extraction step, the total assay time was reduced to 100 min, and the estimated cost per sample was less than €0.8, offering significant time and cost savings (Table 1).

**Table 1.** Key main features of the SYBR Green-based RT-PCR saliva test.

Sensitivity	96%
Specificity	100%
Inactivation time	30 min
Sample processing time	<10 min
Reaction time	60 min
Total assay time	100 min
Cost/sample	<€ 0.8

### COVID-19 mass-testing using the SYBR-Green RT-PCR saliva test

To assess the reliability and usefulness of the SYBR-Green RT-PCR saliva test for community mass screening, we made the test available to students attending public schools in Oeiras (Lisbon district). The target population consisted of children aged 3–12 who had recently become eligible for COVID-19 vaccination (which was ongoing at the time of sampling) and were not being included in the regular testing protocols followed by Portuguese public schools during that period. Over the course of one month, a total of 4445 students underwent testing using the SYBR-Green RT-PCR saliva test, leading to the identification of 80 asymptomatic children whose saliva samples tested positive for SARS-CoV-2 infection. The gold standard test confirmed the infection in all students who had received a positive result from the saliva test. No false positives were reported.

**Figure 4.**
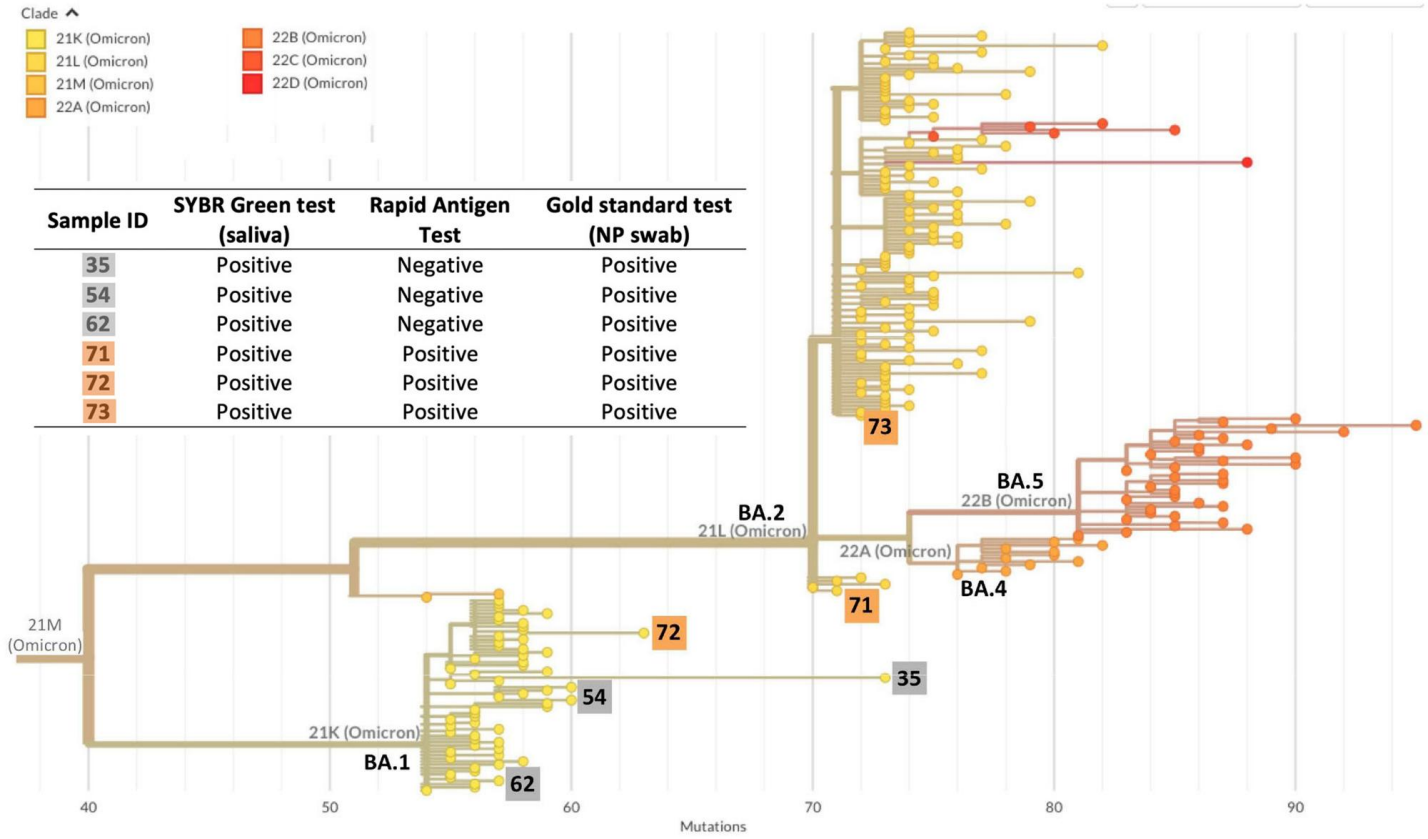
Phylogenetic tree based on the alignment of common SNPs of the Omicron clade highlighting the isolates analysed in this study. Isolates 35, 54, 62, and 72, clustered in clade 21K (BA.1 Pango lineage). Isolates 71 and 73 clustered in clade 21 L (BA.2 Pango lineage). SYBR Green-based one-step RT-PCR saliva assay was in agreement with pharmacy antigen test (RAT) for isolates 71, 72, and 73 (highlighted in orange); SYBR Green-based one-step RT-PCR saliva assay was in disagreement with RAT for isolates 35, 54, and 62 (highlighted in grey). The number of mutations relative to the reference strain used (SARS-CoV-2 Wuhan-Hu-1/2019 virus, GenBank accession number MN908947) is shown below in the xx scale. Tree was generated in Nextclade software (accessed on 04 August 2022).

After receiving the positive result of the saliva tests, 15 children underwent pharmacy rapid antigen tests (RATs) with NP sampling before undergoing gold standard confirmation, with 5 testing negative according to the latter. To determine if specific lineages or variants of SARS-CoV-2 could account for the observed differences in the saliva test and RAT outcomes, we conducted viral genomic sequencing on the RNA extracted from the positive saliva samples. The sequencing analysis was performed on samples where the SYBR-Green RT-PCR results agreed with the RATs (samples 71, 72, and 73), as well as on samples where they disagreed (samples 35, 54, and 62). Genomic sequences were then compared with the genomic diversity of SARS-CoV-2 in Portugal (NextClade database, as of July 2022) to infer phylogenetic relationships. We found that the three viral genomic sequences retrieved from saliva samples of individuals with disparate results between the saliva and antigen tests belonged to sub-variant BA.1 (*n* = 3/3 samples). However, this same sub-variant was identified in samples from infected individuals whose saliva test and antigen tests agreed (sample 72) (Fig. 4). This analysis ruled out the hypothesis that a specific lineage or variant was escaping antigen tests.

## Discussion

The COVID-19 pandemic has raised awareness of the significant importance of diagnosis in assisting the implementation of effective public health measures. However, the gold standard test for COVID-19 was complex and costly, creating challenges for many low- and middle-income countries. This disparity, coupled with limitations in the supply chain of reagents required for the gold standard test, has spurred the development of simpler and more affordable testing options.

Here, we have developed an alternative RT-PCR test based on SYBR Green that demonstrates reliable performance in detecting medium to high viral loads. The test can be conducted directly on saliva specimens or RNA purified from NP swabs. While it is less sensitive than the gold standard test, it exhibits exceptional specificity, making it valuable in scenarios beyond diagnosis. In a pandemic setting, where different testing purposes exist (diagnosis, surveillance, and screening), tests with lower sensitivity or specificity can still play a crucial role [42, 43]. This notion is supported by the successful implementation of public health screenings using less sensitive tests, such as RATs [4]. Indeed, the primary goal of public health screenings is not merely to diagnose individuals but to identify those at higher risk of transmitting the infection, typically those with high viral loads, in order to prevent outbreaks and uncontrolled spread of the disease. This is especially important for COVID-19, as SARS-CoV-2 can be transmitted by presymptomatic or asymptomatic individuals, making symptom-based distancing less effective [44].

In screening scenarios, the speed and frequency of testing are more important than test sensitivity [2, 45]. The cost per test is, consequently, a crucial factor, and our highly specific SYBR Green-based RT-PCR saliva test, costing less than €0.8, offers an interesting alternative to the gold standard. Furthermore, its different chemistry and reagents alleviate the strain on the supply chains associated with RT-PCR testing with fluorogenic probes, ensuring the continuity of diagnosis.

To further reduce testing costs, we have developed a homemade one-step RT-PCR protocol based on EvaGreen. Although its sensitivity is currently inadequate for diagnosis or mass screening purposes, it can serve as a backup option for standard molecular diagnostics in case of supply chain shortages, and it provides a starting point for further optimization.

In our community screening study, the SYBR Green-based direct saliva RT-PCR test demonstrated robust performance. By eliminating the laborious RNA extraction step, the test reduced costs and accelerated result delivery. The test exhibited excellent clinical specificity, correctly identifying 80 infected children confirmed by the gold standard test. Although targeting several genes has been shown to decrease the ratio of false positives [26], using our simple singleplex assay no false positives were observed despite the considerable size of the tested population (4445 tests performed). Interestingly, our test outperformed RATs, as the latter failed to detect 5/15 positive samples. Whole genome sequencing analysis revealed that different SARS-CoV-2 lineages did not account for these discrepancies, suggesting that inefficient NP sampling, typically not always well tolerated by children, or lower sensitivity of the RATs used by some pharmacies at the time may explain the differences.

During the COVID-19 pandemic, multiple variants of the original SARS-CoV-2 strain have emerged as a result of acquired mutations in various regions of the viral genome. Some of these variants were initially identified due to an inability to amplify specific genomic regions using commercial primers, and subsequent confirmation was achieved through whole-genome sequencing [46]. Although not all mutations impact the progression of the pandemic, certain mutations have contributed to the emergence of more transmissible or immune-evading variants [47]. Given the significance of these variants, detecting and monitoring them in the community can be valuable for guiding public health interventions. To the best of our knowledge, there is only one published report where SARS-CoV-2 variant detection was attempted using SYBR-Green-based RT-PCR [48]. The authors used a two-step SYBR-Green RT-PCR protocol with primers targeting the spike and nsp6 gene regions, specifically focusing on the amino acid residue deletions 69/70 and 106/107/108, respectively. In our study, we attempted a similar one-step approach to detect the Δ3675–3677 mutation in the ORF1a region (data not shown), however, in our experiments, we found that to completely eliminate amplification of the original SARS-CoV-2 strain, we needed to employ the allele-specific amplification strategy. Unlike the aforementioned authors, we optimized the SYBR-Green-based RT-PCR protocol for variant discrimination using plasmids (pWT and pVOC), which could simulate higher viral loads and facilitate the detection of less efficient RT-PCR reactions. While we have not attempted to amplify variants carrying single nucleotide mutations, we assume that this is possible using this method, as there are several reports in the literature showing that ASP is capable of discriminating single nucleotide polymorphisms (SNPs) [49–51].

Respiratory viruses have historically been a frequent cause of pandemics [52], and the detection of these virus in saliva has emerged as valuable diagnostic tool [53–58]. In this regard, our saliva test, which can be easily adjusted for other pathogens and is capable of reliably identify (pre-)asymptomatic individuals in community scenarios, certainly offers many benefits in the battle against future silent pandemics.

## Supplementary Material

bpae035_Supplementary_Data

## Data Availability

The datasets generated and analysed during the current study are available in GeneBank repository, with the following accession numbers: OR428193 (sample 35), OR428194 (sample 54), PP755020 (sample 62), OR428195 (sample 71), OR428196 (sample 72), and OR428197 (sample 73).
