# Supplementary material for: A one-step low-cost molecular test for SARS-CoV-2 detection suitable for community testing using minimally processed saliva
Source: Biol Methods Protoc. 2024 May 22;9(1):bpae035. doi: 10.1093/biomethods/bpae035 (PMC11147803; doi:10.1093/biomethods/bpae035)
Supplement: bpae035_Supplementary_Data [file bpae035_supplementary_data.docx]

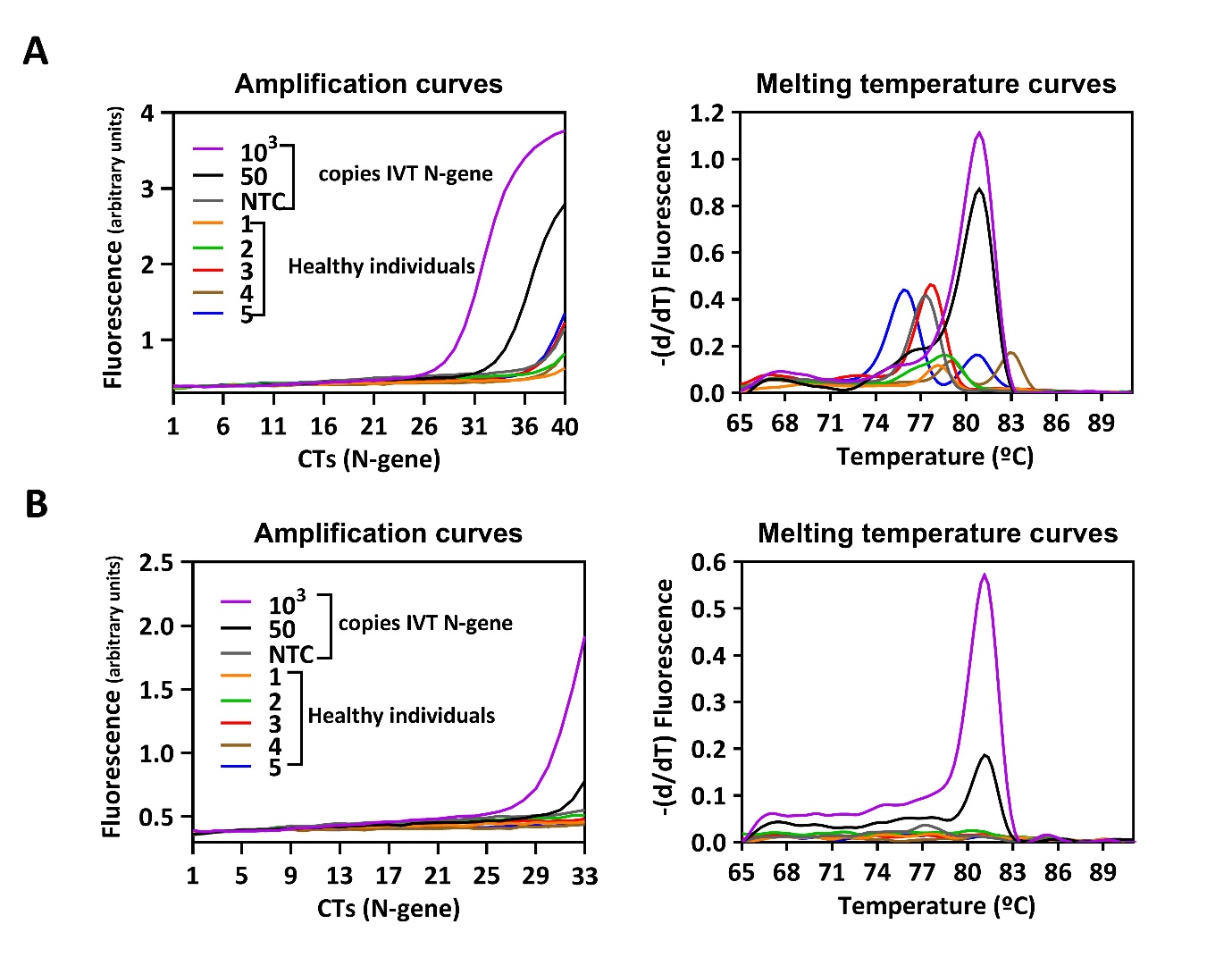


**Figure S1.** Amplification and melting curve profiles after 40 and 33 amplification cycles using the SYBR Green RT-PCR assay. Plots were obtained after **(A)** 40 cycles and **(B)** 33 cycles of amplification of SARS CoV-2 IVT N-gene and RNA extracted from NP swabs obtained from healthy individuals.


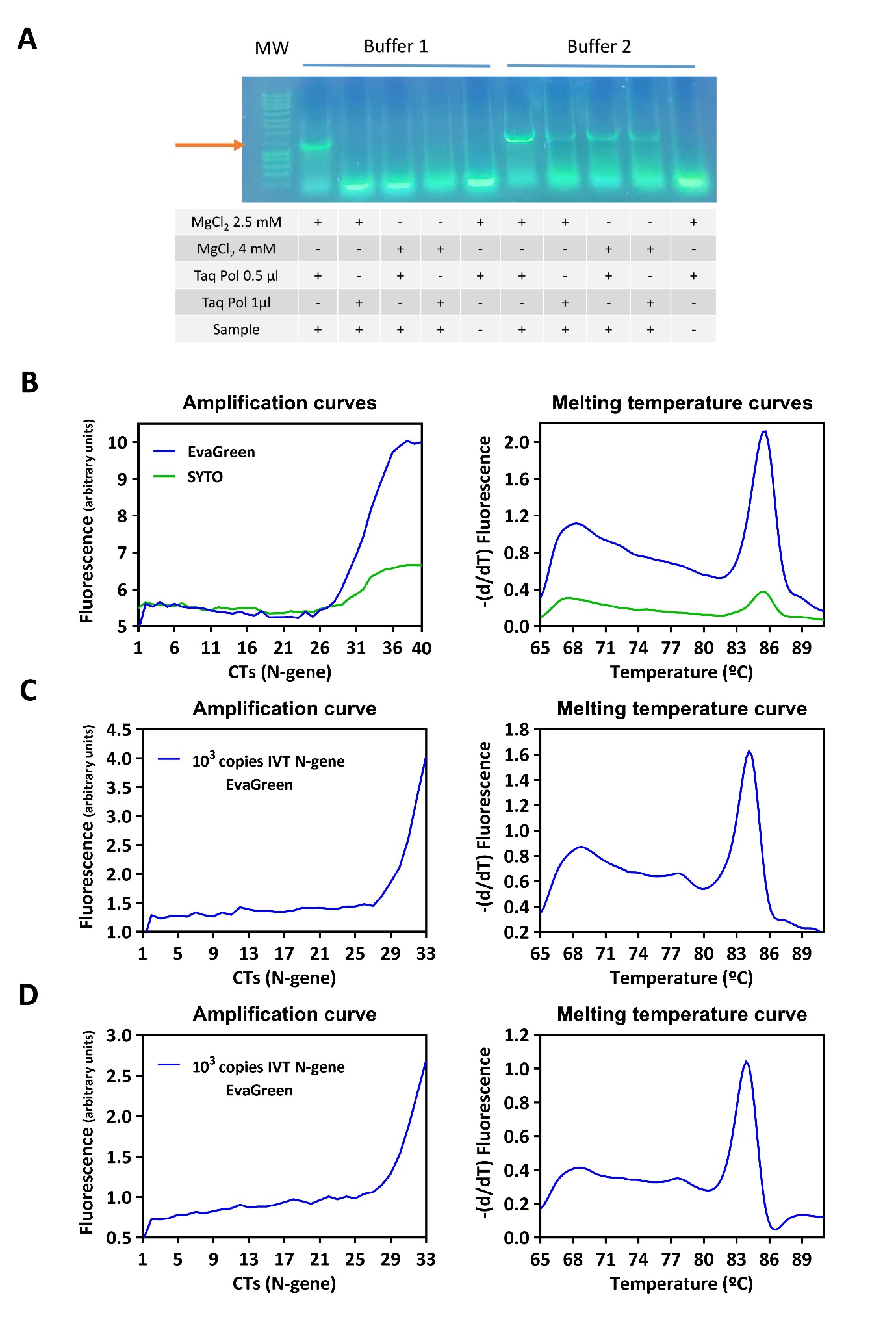


**Figure S2.** Optimization of an in-house made RT-PCR assay using DNA-intercalating dyes. (**A**) Optimization of the PCR reaction conditions using the in-house produced Taq DNA polymerase. Buffer 1 and buffer 2 are two commercial buffers, from Promega (GoTaq polymerase) and NZYTech (NZYTaq II polymerase), respectively. MW, molecular weight marker (NZY DNA Ladder III). (**B**) Amplification and melting curves of 10^3^ copies of IVT N-gene using two intercalating dyes, EvaGreen (Biotium) and SYTO (ThermoFisher). The analysis was performed in the LightCycler 480 (Roche) using the following settings: reverse transcription step (50ºC, 10 min and 95ºC, 3 min), amplification step (40 cycles of 94ºC, 30 s; 58ºC, 30 s and 72ºC, 5 s), melting curve step (95ºC for 5 s; 65ºC for 1 min and 97ºC in continuous mode) and a final cooling step (40ºC, 30 s). (**C**) Amplification and melting curve of 10^3^ copies of IVT N-gene with EvaGreen. The analysis was performed in the LightCycler 480 (Roche) using the following settings: reverse transcription step (50ºC, 10 min and 95ºC, 3 min), amplification step (33 cycles of 94ºC, 30 s; 58ºC, 30 s and 72ºC, 5 s), melting curve step (95ºC for 5 s; 65ºC for 1 min and 97ºC in continuous mode) and a final cooling step (40ºC, 30 s). (**D**) Amplification and melting curve of 10^3^ copies of IVT N-gene with EvaGreen. The analysis was performed in the LightCycler 480 (Roche) using the following settings: reverse transcription step (50ºC, 10 min and 95ºC, 3 min), amplification step (33 cycles of 94ºC, 30 s; 60ºC, 30 s and 72ºC, 5 s), melting curve step (95ºC for 5 s; 65ºC for 1 min and 97ºC in continuous mode) and a final cooling step (40ºC, 30 s).


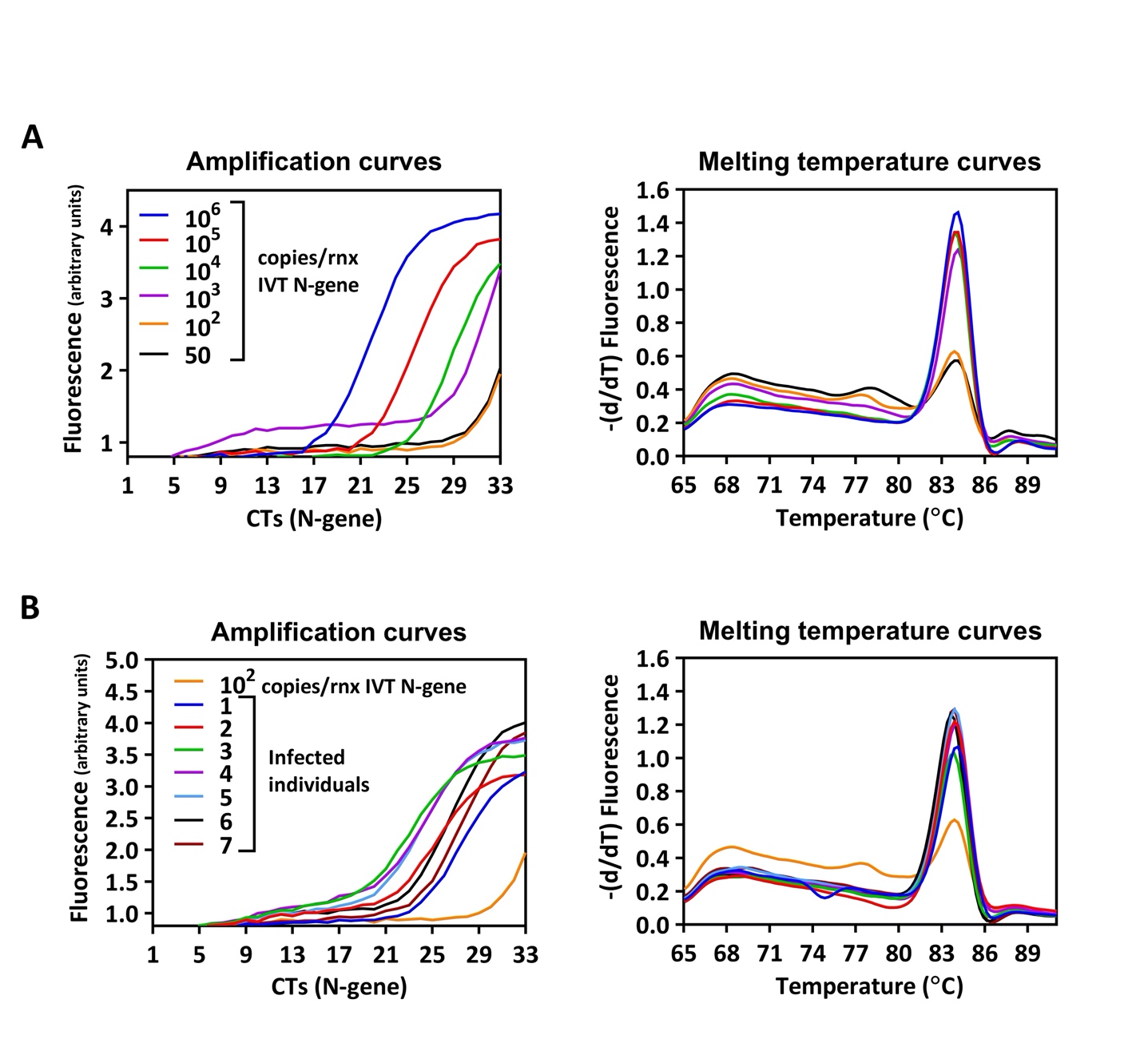


**Figure S3.** Assessment of the in-house EvaGreen RT-PCR test for SARS-CoV-2 detection. Amplification and corresponding melting profiles of the amplicons obtained using copies of (**A**) IVT N-gene or (**B**) RNA isolated from NP swabs of SARS CoV-2 infected patients.


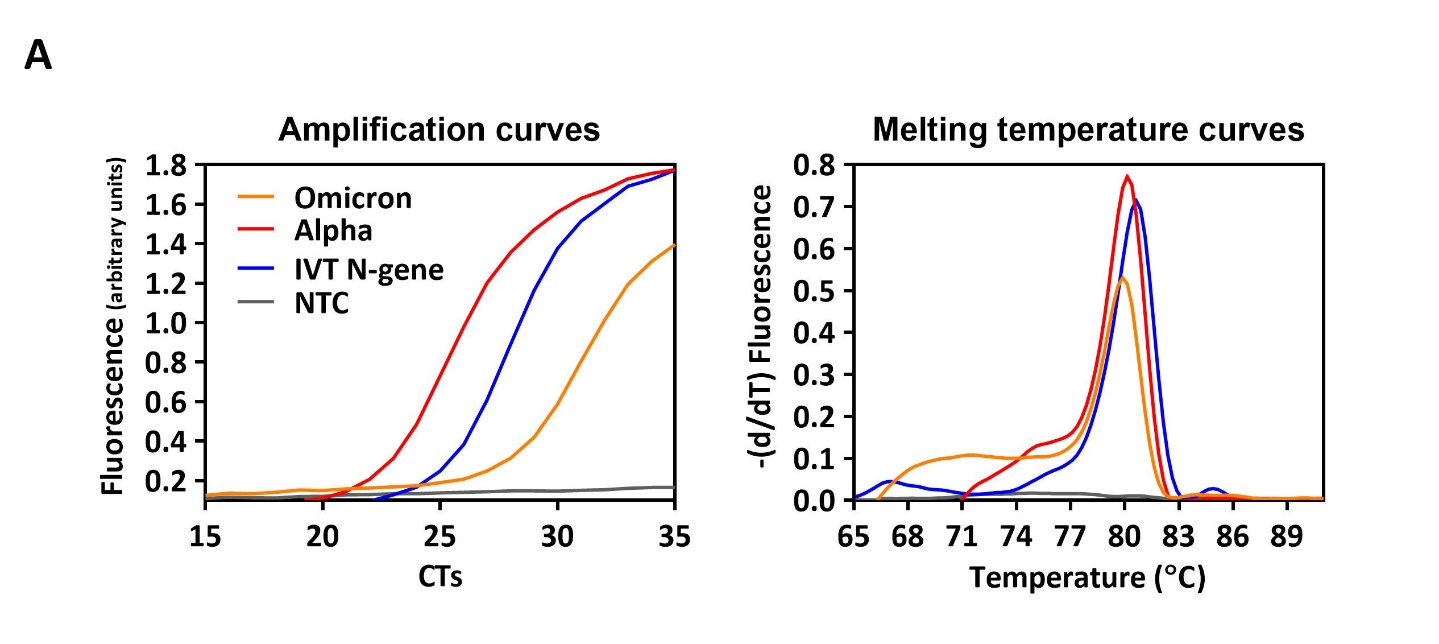


**Figure S4.** Amplification and melting curve profiles of Omicron and Alpha RNA samples obtained with the set of primers used for the detection of the N-gene. 10^4^ copies/rxn of IVT N-gene (blue) were used as a positive control. RNA was extracted from NP swab samples of SARS-CoV2 infected individuals. The analysis was performed in the LightCycler 480 (Roche) and the settings used were the ones used in the detection of ORF1a Δ3675-3677 mutation. Reverse transcription step (50ºC, 10 min and 95ºC, 3 min), amplification step (35 cycles of 94ºC, 30 s; 62ºC, 30 s and 72ºC, 5 s), melting curve step (95ºC for 5 s; 65ºC for 1 min and 97ºC in continuous mode) and a final cooling step (40ºC, 30 s).
